# Transcriptomic analysis of bone transport reveals different functions between both ends

**DOI:** 10.3389/fphys.2025.1592288

**Published:** 2025-05-23

**Authors:** Maochun Wang, Jiao Zhang, Chongxu Qiao, Shunchao Yan, Weicheng Gao, Guoping Wu

**Affiliations:** Department of Plastic Surgery, The Affiliated Friendship Plastic Surgery Hospital of Nanjing Medical University, Nanjing, Jiangsu, China

**Keywords:** bone transport, compressive and tensile ends, transcriptomic analysis, osteogenesis, myogenesis

## Abstract

**Background:**

Bone fractures are common in both young and elderly populations, and bone transport surgery is a critical orthopedic procedure for patients with severe fractures, bone defects, and non-unions. However, the specific molecular mechanisms driving bone healing during bone transport, particularly the roles of compressive and tensile ends, remain poorly understood.

**Methods:**

We utilized transcriptomic analysis of bone tissues from a rat bone transport model to explore differential gene expression patterns associated with compressive and tensile ends.

**Results:**

233 differentially expressed genes (DEGs) were identified in the tensile end (TE) group and 317 DEGs in the compressive end (CE) group, compared to the control group. These DEGs were enriched in distinct biological processes. The TE group was primarily associated with bone healing processes such as ossification, extracellular matrix organization, and bone development. Key genes in the TE group, including Bglap, Acan, Mmp13, and Runx2, were upregulated, highlighting their roles in osteogenesis. In contrast, the CE group showed enrichment in processes related to myogenesis, such as muscle system processes and skeletal muscle tissue development. Core genes in the CE group, including Chrna1, Chrnd, Myod1, and Rps6kb1, were upregulated, indicating a focus on myogenesis and its indirect impact on bone healing. Notably, 15 DEGs were shared between the TE and CE groups, with consistent expression trends, suggesting partially overlapping molecular mechanisms in bone healing under different mechanical ends.

**Conclusion:**

These findings provided insights into the distinct and shared molecular pathways involved in bone regeneration during bone transport and could inform targeted therapeutic strategies to enhance bone healing.

## Introduction

Bone fracture is one of the most common bone related diseases in young and elderly people, particularly in individuals with osteoporosis (Fazzalari, 2011; Wu et al., 2021). It causes immediate physical pain and injury, and can lead to extended periods of work absence, increased disability, impaired mobility and high medical expenses, which imposes substantial economic burdens on both society and families (Iliaens et al., 2021; Singaram and Naidoo, 2019).

Bone transport is one of the important orthopedic surgeries in patients with severe fractures, bone defects and non-unions (Berner et al., 2012; Aktuglu et al., 2019), which is a complex and highly regulated process that plays a critical role in the repair and regeneration of bone tissue following injury or surgical intervention. It involves the controlled movement of bone segments under the influence of mechanical forces, which are essential for bone remodeling and healing. The mechanical environment of bone tissue between two primary ends has distinct effects on cellular behavior and molecular responses within the bone, influencing the rate and quality of bone regeneration. Bone transport promotes the release of small extracellular vesicles rich in miRNAs from local tissues through mechanical stress stimulation, and miR-494-3p has the potential to regenerate multiple tissues (Xie et al., 2024). Local injection of recombinant human BMP-2 or BMP-7 induces bone formation in distraction osteogenesis animal models (Li et al., 2016; Mizumoto et al., 2003). However, the underlying molecular mechanisms that drive bone healing during transport are not fully understood, particularly the distinct roles of compressive and tensile ends on bone tissue.

Transcriptomic studies have emerged as a powerful tool to provide deep insights into the gene expression profiles and regulatory networks involved in bone regeneration during bone transport. Recent studies, particularly using RNA sequencing, have provided the gene expression changes and regulatory networks involved in this process, across the inflammatory, proliferative, differentiation, and remodeling phases (Nakayama et al., 2022; Peng et al., 2018; Liu et al., 2022). The upregulation of genes related to inflammation, cell cycle progression, angiogenesis, osteoblast differentiation, and bone remodeling, as well as the involvement of crucial pathways like Wnt/β-catenin, BMP, and Notch signaling (Chen and Alman, 2009; Schmidt-Bleek et al., 2016; Ballhause et al., 2021; Lin and Hankenson, 2011). These insights highlight the importance of understanding molecular mechanisms to improve surgical outcomes, develop biomarkers for monitoring bone healing, and develop therapeutic strategies to enhance bone regeneration.

In this study, we performed a transcriptome analysis of bone tissue samples collected from rat undergoing bone transport procedures. Our aim was to identify the differential gene expression patterns associated with compressive and tensile ends during the bone transport process. By comparing the transcriptome profiles under these two distinct mechanical environments, we uncovered the molecular mechanisms that drive the differential cellular responses and contribute to the different processes of bone healing. Understanding the distinct functions of compressive and tensile ends on the molecular level may lead to targeted interventions that optimize the mechanical conditions for bone regeneration, ultimately improving clinical outcomes for patients with bone defects or injuries.

## Methods

### Quality control of rat transcriptomes

Transcriptomic data from a rat bone transport model were obtained from Gene Expression Omnibus (GEO) database (GSE200518) (Lin et al., 2023), including six control samples (CTRL = 6, GSM6035372-GSM6035377), three samples with compressive end (CE = 3, GSM6035378-GSM6035380) and three samples with tensile end (TE = 3, GSM6035381-GSM6035383). The rat bone transport model was created with 8 mm bone defect, and 4 mm segmental bone was shaped in rat femurs. Raw data of the transcriptomes was based on Affymetrix Rat Transcriptome Array 1.0, which was read and normalized on Transcriptome Analysis Console software (v4.0.3.14, Thermo Fisher Scientific). The values of the probes were output and re-edited in RStudio (v2023.06.1 Build 524), with probes converted into gene symbols and the expression levels calculated using the average method. All coding genes were extracted with a total number of 20,280. Boxplot of all transcriptome data were plotted using boxplot function in BiocGenerics (v 0.50.0) R package. Principal Component Analysis (PCA) of the rat transcriptome was constructed with factoextra (v1.0.7) and FactoMineR (v2.9) R packages.

### Identification of differentially expressed genes

Compressive end (CE) and tensile end (TE) groups were compared with control (CTRL) groups, respectively, and differentially expressed genes (DEGs) were screened based on 2-fold expression difference and P-value less than 0.05 as the screening criteria. Volcano plot of the two comparisons were created with ggplot2 (v3.5.1) R package and top 5 DEGs of the upregulated and downregulated genes were marked. Top 10 DEGs of the upregulated and downregulated genes were clustered by pheatmap (v1.0.12) R package. All DEGs were further enriched with biological process of Gene Ontology (GO) and Kyoto Encyclopedia of Genes and Genomes (KEGG) with clusterProfiler (v4.12.0) and enrichplot (v1.24.0) R packages. The top 15 biological process terms and top 10 KEGG pathways with the highest number of genes and most significant P-values were displayed.

### Statistical analysis

Venn diagram of shared DEGs in CE and TE groups compared with CTRL group was illustrated with VennDiagram (v1.7.3) R package. Gene relative expression was plotted in GraphPad Prism software (v9.5.1) with unpaired student t test, where * represented statistical differences and ns represented no statistical differences. Protein-protein interaction (PPI) networks for all the DEGs in the CE and TE groups were constructed using the STRING database and Cytoscape software (v3.10.1), respectively.

## Results

### Quality control of transcriptomes in rat bone transport

**FIGURE 1:**
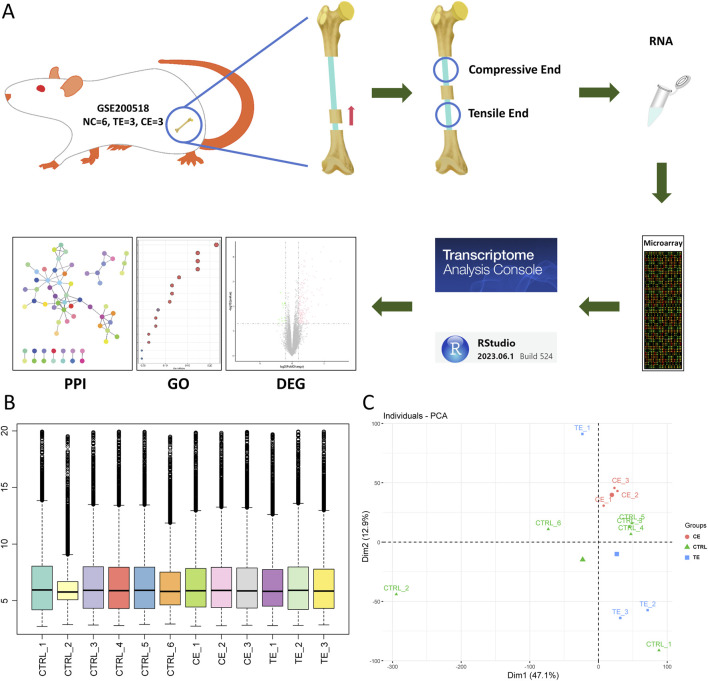
Study overview. **(A)** A schematic of this study. Transcriptome of rat bone transport was obtained with GSE200518, and quality control and analysis of the data were performed and visualized with transcriptome analysis console software and RStudio. **(B)** Boxplot of rat transcriptome after normalization. **(C)** Principal component analysis of all the transcriptome. Circle represented compressive end group, triangle represented control group and square represented tensile end group.

In order to explore the specific molecular mechanisms of compressive and tensile ends on bone regeneration in rats during bone transport, we analyzed the transcriptome of bone tissue in a rat bone transport model (Figure 1A). After normalization with TAC software, all coding genes were extracted from the transcriptome data. Boxplots showed relative consistent median levels across groups, indicating that comparisons between groups could be conducted (Figure 1B). PCA analysis revealed significant differences between the CE group, TE group, and CTRL group, while also showing similarities within each group, particularly in the CE group (Figure 1C). However, two samples deviated from their respective groups, one from the control group and one from the TE group, possibly due to issues in the sampling process or the inherent complexity of bone healing.

### Gene enrichment in tensile end (TE) and compressive end (CE) groups

**FIGURE 2:**
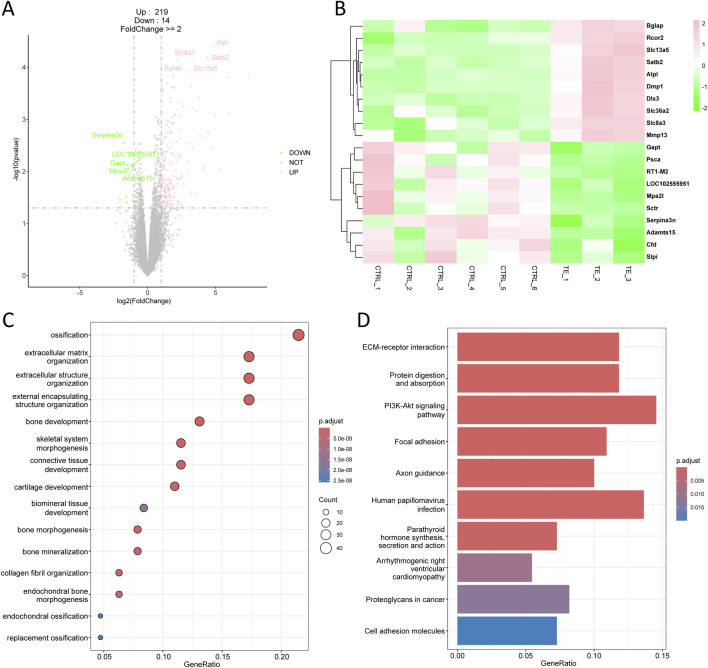
Transcriptomic analysis of tensile end in bone transport. **(A)** Volcano plot of differentially expressed genes in tensile end compared with control group. Pink indicated upregulated genes and green indicated downregulated genes. Top five differentially expressed genes were labeled. **(B)** Heatmap of top 10 differentially expressed genes in tensile end. **(C)** Biological process enrichment of differentially expressed genes in tensile end. **(D)** Kyoto encyclopedia of genes and genomes (KEGG) analysis of differentially expressed genes in tensile end.

In the comparison between TE and CTRL group, 233 differentially expressed genes (DEGs) were identified, comprising 219 upregulated genes and 14 downregulated genes (Figure 2A). The top 10 upregulated genes included Bglap, Rcor2, Slc13a5, Satb2, Alpl, Dmp1, Dlx3, Slc36a2, Slc8a3, and Mmp13, whereas the top 10 downregulated genes included Gapt, Psca, RT1-M2, LOC1022555951, Mpa2l, Sctr, Serpina3n, Adamts15, Cfd, and Slpi (Figure 2B). All the DEGs, including both upregulated and downregulated genes, were used for downstream gene ontology (GO) enrichment and kyoto encyclopedia of genes and genomes (KEGG) analysis. GO enrichment analysis indicated that these DEGs were primarily associated with ossification, extracellular matrix and structure organization, bone development, and bone morphogenesis (Figure 2C), which were biological processes closely related to bone repair. KEGG demonstrated that these DEGs were enriched in pathways such as ECM-receptor interaction, protein digestion and absorption, and PI3K-Akt signaling pathway (Figure 2D).

**FIGURE 3:**
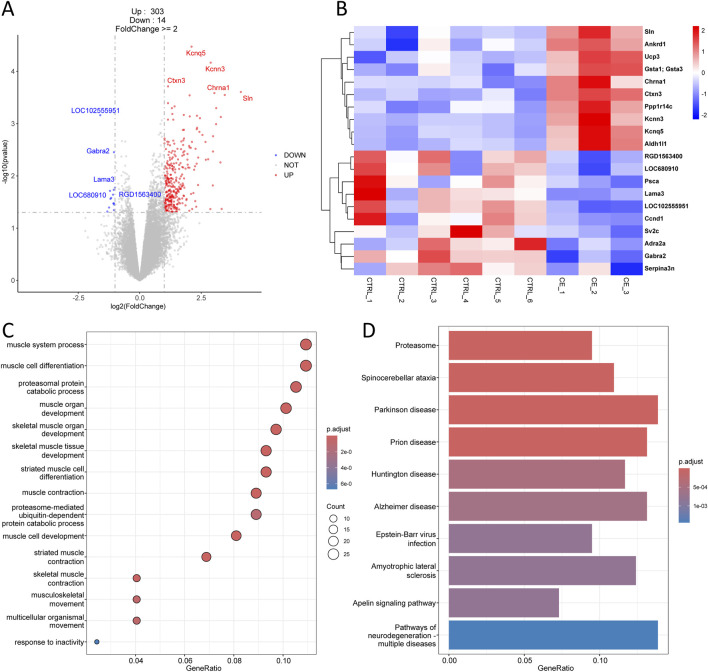
Transcriptomic analysis of compressive end in bone transport. **(A)** Volcano plot of differentially expressed genes in compressive end compared with control group. Red indicated upregulated genes and Blue indicated downregulated genes. Top five differentially expressed genes were labeled. **(B)** Heatmap of top 10 differentially expressed genes in compressive end. **(C)** Biological process enrichment of differentially expressed genes in compressive end. **(D)** KEGG analysis of differentially expressed genes in compressive end.

In the comparison between CE and CTRL group, there were 317 DEGs, including 303 upregulated genes and 14 downregulated genes (Figure 3A). The top 10 upregulated genes comprised Sln, Ankrd1, Ucp3, Gsta1, Chrna1, Ctxn3, Ppp1r14c, Kcnn3, Kcnq5, and Aldh1l1, while the top 10 downregulated genes included RGD1563400, LOC680910, Psca, Lama3, LOC102555951, Ccnd1, Sv2c, Adra2a, Gabra2, and Serpina3n (Figure 3B). All the DEGs, including both upregulated and downregulated genes, were used for downstream GO enrichment and KEGG analysis. Interestingly, the DEGs associated with compressive end were mainly enriched in biological processes related to muscle system process, muscle cell and skeletal muscle tissue development, and muscle contraction (Figure 3C). KEGG analysis showed that these DEGs were primarily associated with proteasome, spinocerebellar ataxia, parkinson disease, and prion disease (Figure 3D). These enrichment results differed from those of tensile end, indicating that compressive and tensile ends had distinct functions during bone transport.

### Shared DEGs in TE and CE groups

**FIGURE 4:**
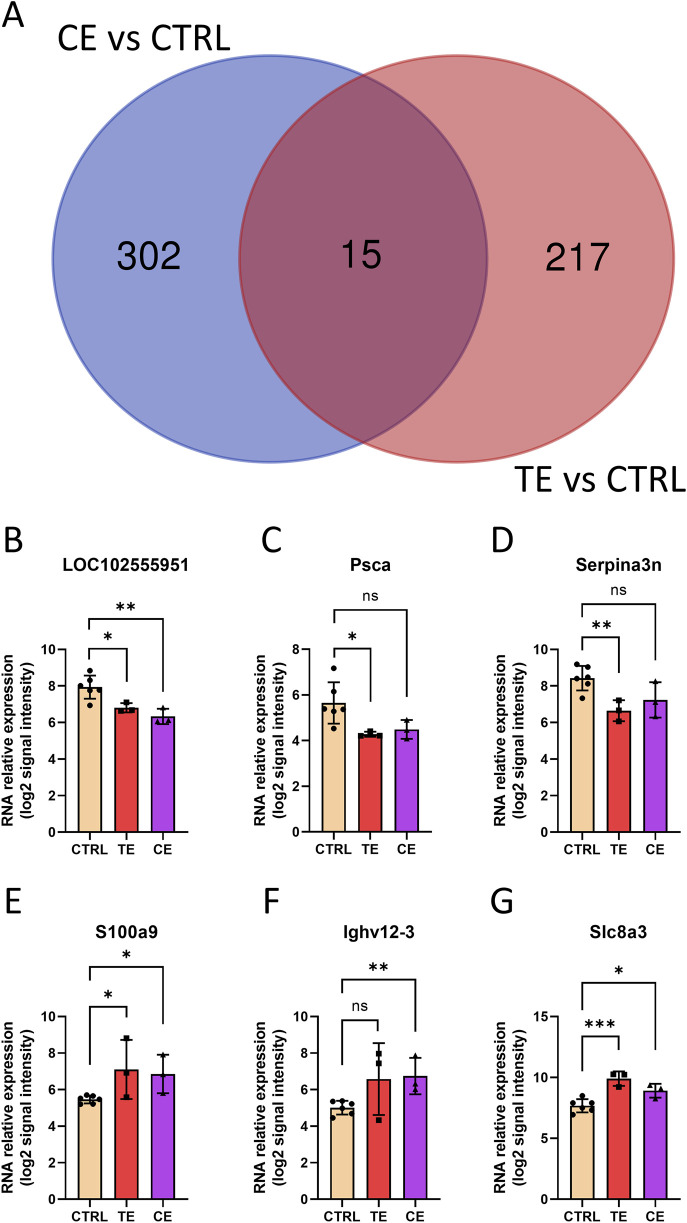
Shared genes in tensile end and compressive end groups. **(A)** Venn diagram of differentially expressed genes in tensile end and compressive end groups. CE, compressive end; TE, tensile end. **(B–G)** Relative expression of shared genes LOC102555951 **(B)**, Psca **(C)**, Serpina3n **(D)**, S100a9 **(E)**, Ighv12-3 **(F)**, and Slc8a3**(G)** in control (n = 6), tensile end (n = 3) and compressive end (n = 3) groups. CTRL, control; CE, compressive end; TE, tensile end. *p < 0.05, **p < 0.01, ***p < 0.001, ns indicated no statistical differences.

Meanwhile, we found 15 common differentially expressed genes in both the CE and TE groups, along with 302 CE-specific genes and 217 TE-specific genes (Figure 4A). Surprisingly, the expression trends of these 15 common DEGs were strikingly consistent, being either upregulated in both CE and TE groups or downregulated in both. For instance, LOC102555951, Psca, and Serpina3n were downregulated in both the TE and CE groups compared to the CTRL group, while S100a9, Ighv12-3, and Slc8a3 were all upregulated (Figures 4B–G). Some genes did not exhibit statistical differences, but they showed similar trend. The consistent expression changes in the TE and CE groups suggested that compressive and tensile ends might share partially identical molecular mechanisms in bone healing. Furthermore, this implied that these genes might play important roles in bone transport, and their interference could potentially contribute to the prognosis of patients after bone transport surgery.

### Molecular network in rat bone transport

**FIGURE 5:**
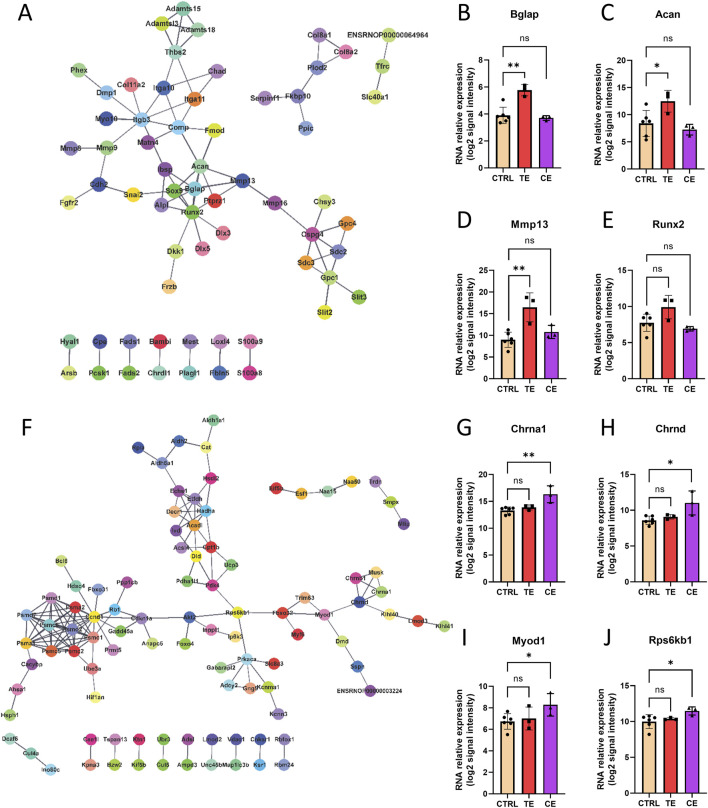
Molecular network of bone transport. **(A)** Protein-protein interaction (PPI) network of tensile end. **(B–E)** RNA relative expression of hub genes Bglap **(B)**, Acan **(C)**, Mmp13 **(D)**, Runx2 **(E)** in control, tensile end and compressive end groups. **(F)** PPI network of compressive end. **(H–L)** RNA relative expression of hub genes Chrna1 **(G)**, Chrnd **(H)**, Myod1 **(I)**, Rps6kb1 **(J)** in control (n = 6), tensile end (n = 3) and compressive end (n = 3) groups. CTRL, control; CE, compressive end; TE, tensile end. *p < 0.05, **p < 0.01, ns indicated no statistical differences.

Finally, we constructed protein-protein interaction (PPI) networks for differentially expressed genes in the TE and CE groups, respectively (Figures 5A,F). We discovered that certain hub genes in the TE group were associated with osteogenesis, including Bglap, Acan, Mmp13, and Runx2 (Figures 5B–E), all of which were upregulated in the TE group with no significant change observed in the CE group. Although Runx2 did not reach statistical significance, it still displayed an upregulated expression trend within the TE group. Conversely, in the CE group, several core genes related to muscle development, including Chrna1, Chrnd, Myod1, and Rps6kb1 (Figures 5G–J), were upregulated in the CE group and showed no significant change in the TE group. These findings indicated that compressive and tensile ends have different molecular mechanisms in bone transport.

## Discussion

Bone transport is a critical technique for addressing large segmental bone defects, with a rich history and ongoing advancements in orthopedics. Since Ilizarov and colleagues first reported the lengthening procedure by applying skeletal traction following osteotomy of the femur, an increasing number of studies have been dedicated to the technique of bone transport (Lin et al., 2023; Ilizarov, 1990; Paley and Maar, 2000). Bone transport is widely applied in the treatment of various complex orthopedic conditions, including infected bone defects, post-traumatic large segment bone defects, and diabetic foot (Rigal et al., 2012; Aktuglu et al., 2019; Ou et al., 2022). However, existing research has primarily focused on clinical applications and the development of various medical materials, with limited investigation into the fundamental mechanisms of bone transport. Here, we utilized transcriptomic data from a rat model of bone transport to reveal the molecular mechanisms by which compressive and tensile ends have both similar and largely different effects on bone healing. The results will provide a theoretical foundation for the study of basic research in bone transport as well as the development of clinical therapeutics.

After quality control of the transcriptomic data, we identified numerous differentially expressed genes in both the TE (tensile end) group and the CE (compressive end) group. The number of upregulated genes was greater than that of downregulated genes, with 219 upregulated genes in the TE group and 303 in the CE group, while both groups had 14 downregulated genes. This implies an increased activity in gene expression during the bone transport process to accomplish the healing of bone tissue. These results may also vary due to factors such as different studies, types of samples, technologies used, or the methods of analysis.

We found many identical differentially expressed genes in the TE group and the CE group, including LOC102555951, Psca, Serpina3n, S100a9, Ighv12-3, and Slc8a3, etc. Most surprisingly, they all showed the same trends of upregulation or downregulation in both the TE and CE groups. This suggested that compressive and tensile ends have some of the same molecular mechanisms for bone healing during the bone transport process. S100a9 is a calcium-binding protein associated with inflammation and immune responses. Studies have indicated that S100a9 was involved in the regulation of the inflammatory response (van den Bosch et al., 2016; Wang et al., 2018), which is crucial for bone repair. Serpina3n is a serine protease inhibitor involved in the regulation of tissue remodeling and inflammatory responses (Hsu et al., 2014; Ishida et al., 2018). Its role in bone healing may involve protecting tissues from excessive degradation and promoting the stability of the healing environment. Slc8a3 is a sodium-calcium exchanger that plays a key role in maintaining the balance of intracellular calcium ions. Calcium signaling is very important in the differentiation of osteoblasts and the bone healing process (Aquino-Martínez et al., 2017), and Slc8a3 may affect these processes by regulating calcium ion concentrations. Psca is a cell surface antigen, with most research focusing on its role in cancer (Yang et al., 2014), but some studies suggest it may also play a role in cell adhesion and signal transduction (Li et al., 2017; Bahrenberg et al., 2001), which could indirectly contribute to the bone healing process.

Meanwhile, the differentially expressed genes in the TE group and the CE group were enriched in different biological processes. The TE group was mainly related to bone healing, including ossification, extracellular matrix and structure organization, bone development, and bone morphogenesis. This was consistent with the reported mechanisms of distraction osteogenesis, which were mainly related to ossification and extracellular matrix. Several core genes in the molecular network of the TE group were also related to bone healing, which were upregulated in the TE group and unchanged in the CE group. For example, Bglap gene encodes osteocalcin, a non-collagen protein secreted by osteoblasts and one of the main components of the bone matrix. Osteocalcin plays an important role in the process of bone mineralization and is also involved in regulating the mineral balance (Berezovska et al., 2019; Manolagas, 2020). Acan gene encodes aggrecan, which is mainly found in cartilage tissue and is an important part of the cartilage matrix (Roughley and Mort, 2014), supporting the formation of the cartilage matrix during endochondral ossification. Mmp13 gene encodes matrix metalloproteinase 13, an important protease that can degrade a variety of extracellular matrix proteins, especially type II collagen. Mmp13 promotes the migration of osteoblasts and the formation of new bone by degrading cartilage matrix, playing a key role in bone remodeling and healing (Paiva and Granjeiro, 2017; Tang et al., 2012). Runx2 is the master regulatory factor for bone formation, regulating the differentiation of osteoblasts and the expression of bone matrix proteins, and plays a central role in bone development and osteoblast differentiation (Komori, 2010). In contrast, the CE group showed many different biological processes from the TE group, mainly related to myogenesis, including muscle system process, muscle cell and skeletal muscle tissue development, and muscle contraction. Several core genes in the molecular network of the CE group were upregulated in the CE group and unchanged in the TE group. For example, Chrna1 and Chrnd encode different subunits of the nicotinic acetylcholine receptor, mainly related to neuromuscular function (Liao et al., 2022; Vogt et al., 2008), which may indirectly affect bone healing by affecting the function of surrounding muscles. Myod1 is a key muscle-specific transcription factor that plays a key role in muscle regeneration and may indirectly promote bone healing by promoting muscle repair (Goudenege et al., 2009). Rps6kb1 encodes the ribosomal protein S6 kinase B1, a key regulatory factor of the mTOR signaling pathway, directly involved in the proliferation of osteoblasts and the synthesis of bone matrix, and is an important gene in the process of bone healing (Li et al., 2024; Tang et al., 2021). These results indicated the different molecular mechanisms of compressive and tensile ends during bone transport process.

This study also had some limitations. First, these transcriptomes were based on the coding genes of chip platform, which might miss non-coding genes with important functions. Using RNA sequencing technology could uncover more valuable information. Second, the small sample size and the differences between samples may affect the results. Small sample size and limited sample replication may lead to false positives and the loss of some false negatives. Future studies need to increase sample replication to remedy this shortcoming and make the results more accurate. Third, further functional validation of these genes in molecular networks is needed to enhance the significance of the findings. Last, future research should integrate transcriptomics with other omics approaches to provide a comprehensive understanding of bone regeneration and support the development of personalized treatments for patients undergoing bone transport surgery.

## Conclusion

In conclusion, our study delineated the unique and shared molecular pathways influenced by compressive and tensile ends in bone transport, offering valuable insights for developing targeted therapeutic strategies to enhance bone healing. The identification of key genes and pathways could potentially lead to improved outcomes in bone transport surgery by modulating these molecular mechanisms.

## Data Availability

The datasets presented in this study can be found in online repositories. The names of the repository/repositories and accession number(s) can be found in the article/supplementary material.
